# *Escherichia coli* Shiga Toxins and Gut Microbiota Interactions

**DOI:** 10.3390/toxins13060416

**Published:** 2021-06-11

**Authors:** Kyung-Soo Lee, Yu-Jin Jeong, Moo-Seung Lee

**Affiliations:** 1 Environmental Diseases Research Center, Korea Research Institute of Bioscience and Biotechnology, 125 Gwahak-ro, Daejeon 34141, Korea; dlrudtn2324@kribb.re.kr; 2 Department of Biomolecular Science, KRIBB School of Bioscience, Korea University of Science and Technology (UST), 127 Gajeong-ro, Yuseong-gu, Daejeon 34113, Korea

**Keywords:** Shiga toxins, Shiga toxin types 1 and 2, Shiga-toxin-producing *Escherichia coli* (STEC), commensal microbes, bacterial toxins, gut microbiota, hemolytic uremic syndrome (HUS)

## Abstract

*Escherichia coli* (EHEC) and *Shigella dysenteriae* serotype 1 are enterohemorrhagic bacteria that induce hemorrhagic colitis. This, in turn, may result in potentially lethal complications, such as hemolytic uremic syndrome (HUS), which is characterized by thrombocytopenia, acute renal failure, and neurological abnormalities. Both species of bacteria produce Shiga toxins (Stxs), a phage-encoded exotoxin inhibiting protein synthesis in host cells that are primarily responsible for bacterial virulence. Although most studies have focused on the pathogenic roles of Stxs as harmful substances capable of inducing cell death and as proinflammatory factors that sensitize the host target organs to damage, less is known about the interface between the commensalism of bacterial communities and the pathogenicity of the toxins. The gut contains more species of bacteria than any other organ, providing pathogenic bacteria that colonize the gut with a greater number of opportunities to encounter other bacterial species. Notably, the presence in the intestines of pathogenic EHEC producing Stxs associated with severe illness may have compounding effects on the diversity of the indigenous bacteria and bacterial communities in the gut. The present review focuses on studies describing the roles of Stxs in the complex interactions between pathogenic Shiga toxin-producing *E. coli*, the resident microbiome, and host tissues. The determination of these interactions may provide insights into the unresolved issues regarding these pathogens.

## 1. Introduction

Numerous supportive public health measures have led people to erroneously believe that epidemics of many bacterial infectious diseases are no longer a serious health risk. However, Shiga-toxin-producing *Escherichia coli* (STEC) still poses a threat to public health. Shiga toxin (Stx) is prototypically synthesized by the bacterium *Shigella dysenteriae* serotype 1, with genetically and structurally related toxin variants produced by certain serotypes of *E. coli*, including enterohaemorrhagic strains of *E. coli* (EHEC) [1]. Bacillary dysentery due to infection by Stx-producing bacteria, characterized by acute infectious diarrhea, primarily affects children aged <5 years [2]. Endemic bacillary dysentery occurs globally, including in portions of Africa, Southeast Asia, and the Indian subcontinent, with an estimated 2–7 per 1000 children per year requiring clinical care and 164,300 deaths per year attributable to shigellosis [3]. By contrast, STEC-associated illnesses in young children are more prevalent in developed countries, in which residents consume higher levels of beef and beef products [4,5]. Major outbreaks of diarrheal diseases caused by EHEC may be due to the ingestion of foods such as uncooked meat, unpasteurized milk, water contaminated with these bacteria, and by the contamination of foods used in the preparation of fast-food [6]. Perhaps the largest outbreak of hemorrhagic colitis was caused by an O157 infection in and around Sakai City, Japan, in 1996, which resulted in approximately 1000 hospitalizations among 7000 infected cases [7]. Many non-O157 STEC serotypes have been increasingly reported, and a massive outbreak caused by the hybrid STEC/enteroaggregative *E. coli* (EAEC) O104:H4 strain occurred in northern Germany from May to June 2011 [8]. More recently, 439 outbreaks and 5 deaths caused by EHEC-contaminated romaine lettuce were reported in multiple states of the United States in 2018 [9].

Stx-producing bacteria have received substantial attention as emergent pathogens due to the dangerous toxins they produce. These exotoxins are the principal virulence factors associated with the pathogenesis of bloody diarrheal diseases, bacillary dysentery, and hemorrhagic colitis progressing to acute renal failure in infected patients, primarily in children. This phenomenon, collectively referred to as hemolytic uremic syndrome (HUS), is the leading cause of pediatric acute renal failure in many countries, including countries in the European Union and the United States [10,11]. Both Stxs and the inflammatory innate immune cells activated by these toxins contribute to the pathogenesis of HUS by rendering blood vessels in the colon, kidney, and central nervous system (CNS) more sensitive to the detrimental action of Stxs. Studies in animals found that treatment with purified Stxs induces intestinal and renal epithelial and endothelial cells to express neutrophil and monocyte chemoattractants. This, in turn, induces the infiltration of peripheral blood mononuclear cells (PBMCs) into the lamina propria and kidneys [12,13]. These findings suggest that the infiltration of inflammatory cells into sites of toxin-induced damage causes the localized expression of cytokines, which in turn facilitate vascular damage via immunopathological reactions.

Studies on Stxs-induced host signaling pathways have indicated that these toxins, which act as multifunctional bacterial proteins, promote ribotoxic stress, apoptosis, endoplasmic reticulum (ER) stress, inflammatory responses, and autophagy in host cells [14]. In addition to the toxigenic and immunopathological potential of Stxs in patients, these toxins interact with multiple cell types in vitro and are responsible for the pathogenic characteristics of HUS in animal models. Although the Stxs-mediated pathogenesis of HUS is not fully understood, comprehensive knowledge of the role of Stxs in altering the composition (also referred to as ‘dysbiosis’) of the intestinal microbiota in a host infected with EHEC compared with a healthy control must be used to help identify the host factors or the commensal microbial-derived products that exacerbate tissue damage or protect against intoxication caused by toxin-producing bacteria. Studies have evaluated the precise correlations between Stx-mediated pathogenesis and intestinal indigenous commensal microbes. This review summarizes the current understanding of the roles of *E. coli* Stxs and STEC at their interfaces with commensal microbiota in the gut, mainly focusing on interactions with human microbiota.

## 2. Toxins

**Figure 1:**
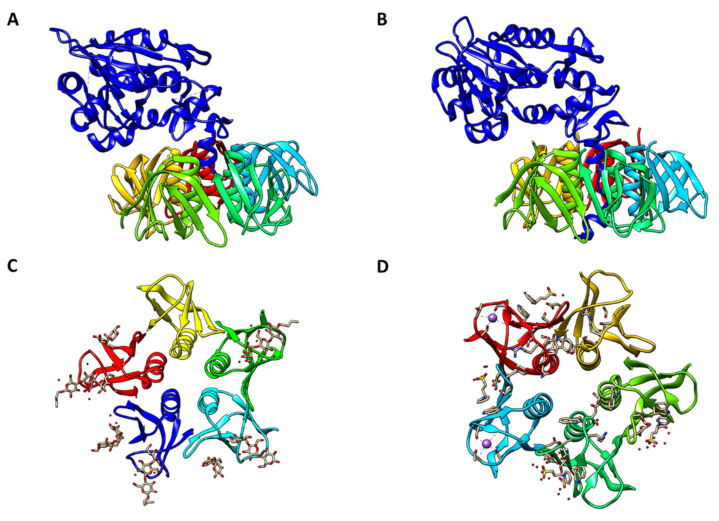
Crystal Structure of Shiga toxin. (**A**) Shiga Toxin Type 1 (PDB #1DM0). (**B**) Shiga Toxin Type 2 (PDB # 1R4P). (**C**) Shiga Toxin 1 B-subunit with Gb3 receptor (PDB #1BOS). (**D**) Shiga Toxin 2 B-subunit with Gb3 receptor (PDB #1R4P, deletion of A-subunit). PDB files of all structures were obtained from RCSB PDB (www.rcsb.org) and PDB files were compiled with Chimera 1.10.2 (UCSF Chimera, www.cgl.ucsf.edu/chimera, accessed on 10 June 2021). Reproduced from reference [14]. 2016, MDPI.

**Figure 2:**
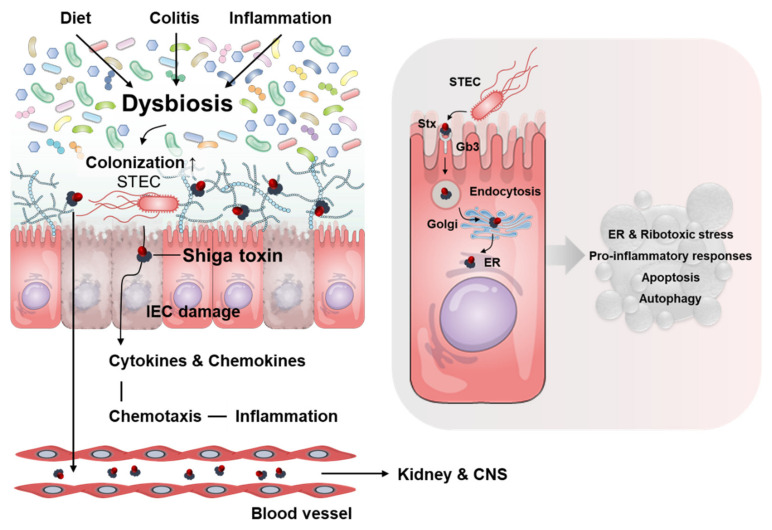
Summary of STEC/Stx-induced immunopathology. Dysbiosis, induced for a variety of reasons such as diet, colitis, and inflammation, increases STEC infection and colonization. STEC induces the delivery of Shiga toxins and the production of cytokines and chemokines through colonization in intestinal epithelial cells. In addition to cell death by Stxs, various cells, including neutrophils induced by chemotaxis, induce inflammation in the intestine, which leads to damage. Toxins pass through the intestinal mucosa, enter the bloodstream and travel to target organs such as the kidneys and CNS. After membrane invasion-mediated endocytosis through the toxin receptor Gb3 on the cell surface, Stxs migrate to the Golgi and ER. Shiga toxin acts as a multifunctional bacterial protein, promoting ER stress, ribotoxic stress, pro-inflammatory responses, apoptosis, and autophagy in host cells.

Stxs produced by bacteria, including EHEC and *Shigella dysenteriae* serotype 1, act as primary virulence factors. Each ribosome-inactivating holotoxin possess an AB5 molecular configuration, as confirmed by high-resolution structural analysis, consisting of a large monomeric 32 kDa A subunit and small homo-pentameric 7.7 kDa B subunits [15] (Figure 1). The enzymatic subunit A of the holotoxin is associated with cytotoxic activity, whereas subunit B binds to host receptors on cell surfaces. Stxs bind with high affinity mainly to the glycolipid Gal (α1→4)Gal (β1→4)Glc (β1→1) ceramide (globotriaosylceramide or Gb3), or to a lesser extent, GalNAcβ1-3Galα1-4Galβ1-4GlcβCer (globotetraosylceramide or Gb4) or Galβ1-3GalNAcβ1-3Galαl-4Galβ1-4Glcβl-lCer (globopentaosylceramide or Gb5), on the host cell surface, which act as the toxin receptors [16,17]. In particular, the B-subunits mediate the specific binding of toxins to target cell receptors and their receptor-mediated uptake into target cells [18]. Of note, as a Gb3-independent Stx-binding receptor, Brigotti et al. demonstrated that the Toll-like receptor 4 (TLR4), a well-characterized pattern recognition receptor for lipopolysaccharide, binds to the toxin on the surface of human neutrophils, which are lacking Gb3, without the internalization or intracellular processing of the toxin [19]. Many different cell types, including human renal proximal tubular epithelial cells, human brain microvascular endothelial cells, and primary human monocyte-derived macrophages, express functional Gb3 on their membrane and therefore are target cells for the binding of Stxs (reviewed in [20]). Following the binding of Stxs to their receptors on host cell surfaces, these toxins may be endocytosed via clathrin-coated pits into early endosomes. Alternatively, Stxs may cluster at the plasma membrane, followed by their uptake by a clathrin-independent mechanism [21,22]. Otherwise, Stxs are unable to enter the cell cytosol directly. A recent, extensively updated review of Stxs-toxin receptor interactions on host cell surfaces provided a concise summary and new insight into receptor analog-mediated therapeutic approaches against bacterial verotoxins and Stxs-mediated cytotoxicity [23]. Following its binding to Gb3 on the cell surface, the holotoxin is internalized via receptor-mediated endocytosis and trafficked intracellularly to early endosomes, the *trans*-Golgi network through the Golgi apparatus, and the lumen of the ER by a process called retrograde transport to deliver Stxs. The precise mechanisms underlying the retrograde transport of Stxs remain largely unexplored, although it occurs in a KDEL-receptor-independent manner [24]. During the intracellular processing of the toxin, subunit A is cleaved by the host protease furin into two fragments: A1, the catalytically active fragment with N-glycosidase activity; and A2, which remains covalently associated through a disulfide bond and is essential for the assembly of the AB5 configuration [15]. Following the reduction of the bond, the A1 fragment retrotranslocates across the ER membrane into the cytosol by utilizing host cellular endoplasmic reticulum-associated protein degradation (ERAD) machinery. The integral membrane Sec61 complex translocon core unit may be involved in the translocation of the A1 subunit. The A1 subunit, which has RNA N-glycosidase activity, inactivates the 60 S subunit of host cell ribosomes by cleaving the N-glycosidic bond at a single specific adenine residue in 28S rRNA (in rats, A4324), leading to the inhibition of the EF-1–dependent aminoacyl-tRNA binding and ultimately preventing aminoacyl-tRNA from binding to the ribosome. This holotoxin subsequently enters the host cell cytosol, leading to multiple cellular responses, including the inhibition of protein synthesis, apoptotic cell death, ER stress, autophagy, and inflammation [25,26,27] (Figure 2). As an alternative way of transporting Stxs into host cells, Stx-containing microvesicles, derived from the plasma membrane of host blood cells in the circulation, reach the kidneys and the toxin is transferred to the renal target cells, including the glomerular endothelial cells, where it is released from the microvesicles and elicits cytotoxic effects by reaching the ribosomes and inhibiting protein synthesis [28]. During STEC-associated HUS, Stxs directly activate complements by triggering a cascade of signaling events and delaying the cofactor activation of surface adhesion factor H (FH) bound to the toxin, leading to the complement-mediated hemolysis with the release of microvesicles from the fragmented red blood cells [29,30]. Buelli et al. reviewed the etiology of complement activation in various experimental models and HUS patients [31]. However, due to the complex pathophysiological cascade of events that ultimately leads to the clinical symptoms of Shigatoxemia, there are no satisfactory therapeutic interventions or regimens for treating children infected with EHEC.

Two major structural types of Stxs have been identified to date: Shiga toxin type 1 (Stx1) and Shiga toxin type 2 (Stx2). Each of these is further subdivided into subtypes, Stx1a, Stx1c, and Stx1d; and Stx2a, Stx2b, Stx2c, Stx2d, Stx2e, Stx2f, Stx2g (reviewed in [32,33]), Stx2h [34], and Stx2i [35,36]. Each subtype consists of a number of variants, which are released by Stx subtype-specific STEC strains. The overall amino acid identity of Stx1 and Stx2 is only 56%, although residues involved in enzymatic activity and binding to cells are more highly conserved. By contrast, variations within the Stx1 and Stx2 categories are much lower, with members having 84–99% amino acid identity. Because the Stx2 variants differ from one another to a greater extent than the Stx1 variants, they have different levels of toxicity in animal models of disease and have different receptor preferences [37]. More importantly, the Stx1a and Stx2a subtypes most commonly cause severe pathogenesis in humans.

The multiple functions of Stxs have been extensively investigated, both in vitro and in vivo, by our group and others. Over the course of these studies, we have devised methods of interrupting Stx-induced host injury signaling mechanisms activated by apoptosis via ER stress, autophagy, or pro-inflammatory cytokine/chemokine production. However, the precise pathogenic potential roles of EHEC-Stxs and other commensal microbiota have not yet been completely investigated. These interactions may be a crucial step in determining the yet unknown mechanisms by which the toxin encounters host microbiota and crosses the mucosal layer.

## 3. Crosstalk with Gut Microbiota in Intestinal Pathology upon STEC Infection

Approximately 10^14^ bacteria, consisting of 500 to 1000 species, are present in the gastrointestinal (GI) tract of a human adult. This population of very diverse bacteria, called the gut microbiota, includes protective bacteria, as well as bacteria that could potentially be harmful to, but maintain a symbiotic relationship with, the host. Cross-regulation between the host and the gut microbiota maintains a homeostatic balance of bacteria, keeping the GI tract healthy and preventing an overgrowth of potentially pathogenic bacteria [38,39]. The Firmicutes, Bacteroidetes, Actinobacteria, and Proteobacteria groups make up the largest share of the normal gut microbiota [40]. Dysbiosis refers to an imbalance in the qualitative and quantitative composition and metabolic activity of intestinal microbiota and may be associated with a variety of disorders including inflammatory bowel disease (IBD), allergies, diabetes, obesity, and multiple sclerosis [41]. Dysbiosis may increase the risk of various gut bacterial infections and has also been linked to STEC infection [42]. Changes in gut microbiota according to diet are well-known [43,44], and Zumbrun et al. reported that dietary choice modulates susceptibility to STEC infection. Mice that were fed a high fiber diet (HFD) exhibited a decrease in native Escherichia species and increased colonization of STEC, weight loss, and mortality compared with mice that were fed a low fiber diet (LFD) [45]. A colitis murine model generated using dextran sulfate sodium similarly had an increased risk of STEC infection [46]. These reports indicate that the robustness of the gut microbiota plays an important role in the defense against STEC infection (Figure 2). Several case reports demonstrated that STEC infection and disease are more likely to occur in children than in adults [47,48]. The gut microbiota of children, which is less mature than that of adults, has a low diversity and number of communities [49]. This is likely one reason for the high rate of STEC infections in children because STEC may colonize individuals with weak/non-healthy microbiota.

During intestinal infection, STEC can reach the ileum and colon and cause disease through survival and colonization [50,51]. STEC colonization is a type of attaching and effacing (A/E) lesion that relies on the type three secretion system (T3SS) to transfer effector proteins to host cells and on the apoptosis of enterocytes located on the apical surface of the intestines. STEC attachment is accompanied by the localized destruction of microvilli [52,53]. Bacteria closely attach to intestinal cells via T3SS and a series of effector proteins encoded in the locus of enterocyte effacement (LEE). Various effector proteins help to sustain the presence of bacteria in the intestinal tract, as well as playing a pivotal role in the toxic effects of these bacteria [54]. After colonization, Stxs produced by STEC pass through intestinal epithelial cells (IECs) into the bloodstream, allowing them to reach target organs, including the kidneys, brain, and eyes, and causing diseases such as HUS [55].

STEC-associated immune responses of the host can include the overstimulation of proinflammatory cytokine production, immune cell activation, and complement activation by Stxs, resulting in primary tissue injury [56,57,58,59]. A continuum of events can result in the development of HUS. Stxs may bind to IECs and mediate the translocation of toxin molecules to and through the basolateral membrane. STEC strains and non-pathogenic commensal *E. coli* showed differential inflammatory responses using an in vitro IEC infection model system [60]. Although no single transcriptional or cytokine response pattern was reported to be characteristic of the early stages of STEC infection, STEC strains and commensal *E. coli* differed significantly in the expression of genes involved in amino acid biosynthesis and in uptake and respiration. These three classes of hypothetical genes were found in a fairly high percentage of other STEC pathotypes [60].

Although STEC infection can occur in a variety of animals, cattle are the main reservoir [61]. STEC colonizes the recto-anal junction (RAJ) in cattle but is asymptomatic [62,63]. This is because the Gb3 expression is lower than in humans, and there are reports that even when Gb3 is expressed in the kidneys and brain, Stxs cannot bind to blood vessels in the bovine GI tract [64]. To colonize the RAJ, STEC uses an acid resistance (AR) system. Glutamate decarboxylase GadA and GadB consume protons by converting glutamate into gamma-amino butyric acid (GABA), which helps to protect cells against acidic stress in the GI tract [65,66]. The LuxR homologue SdiA detects acyl-homoserine lactone (AHL) produced by other bacteria and induces a quorum-sensing (QS) system [67]. STEC detects AHL through SdiA in the rumen, inhibits LEE expression, and activates the GadA/B-mediated AR system to survive in an acidic environment. STEC migrates to the RAJ, and LEE is expressed in the absence of AHL, allowing for the colonization of the GI compartment [66]. *E. coli* O157:H7 in feces is defined as a super-shedder (SS) at a level higher than 10^4^ CFU per gram [68]. Several studies have compared changes in the microbiota between non-shedders (NS) and SS. STEC infection alters the abundance and diversity of some gut microbiota in cattle [69,70]. A paper reviewing data on changes of the microbial count in cattle due to a recent STEC infection classified and organized the microbial differences [71]. The composition of the gut microbiota has been reported to be clinically altered. Lower numbers of *Bifidobacteriales* and *Clostridiales* were found in the feces of patients infected with STEC O26:H11 than in the healthy subjects [72]. Bifidobacteria are involved in the NF-κB and SOCS signaling pathways in IEC lines by downregulating the mRNA levels of inflammatory cytokines in response to stimulation with intact bacterial cells or bacterial cell wall components such as LPS [73,74]. Moreover, Bifidobacteria have been reported to have protective efficacy in mice infected with EHEC O157:H7 [75]. Many studies have demonstrated that *Clostridium* species are probiotics that control the intestinal inflammatory response caused by LPS, suggesting that they have preventive and therapeutic effects on EHEC infection [76,77,78]. Taken together, these findings suggest that STEC infection through intestinal colonization may affect the microbial composition and abundance, which may increase the risk of infection.

In addition to its role in the effects of STEC, Stx is a virulence factor that alone has the potential to affect intestinal tissue damage and microbiota. Clinically, STEC virulence genes, including the *stx2a* and *eae* genes that encode Stxs, have been detected in the feces of STEC-infected patients through meta-genomic analysis [72]. This suggests that Stx not only works by migrating to the lamina propria but can also act in the intestinal lumen. Purified Stx proteins induce ribotoxic stress, apoptosis, and inflammatory responses in a variety of cells. Stxs released after STEC colonization bind to intestinal epithelium but not to normal intestinal cells that do not express Gb3 [79,80]. The human IEC lines Caco-2 and HEp-2 cells, which express Gb3, were sensitive to Stxs, as shown by the induction of apoptosis through ER stress [81,82]. Although fully differentiated T84 cells do not express Gb3 and are resistant to Stx [82], the long-term exposure of T84 cells to Stx2 during in vitro organ culture (IVOC) induces internalization and damage [83]. Dysbiosis due to EHEC colonization of the intestine is also affected by Stxs. For example, Stx2 may be involved in increasing the colonization capacity of EHECs by increasing the expression of necleolin in HEp-2 cells [84]. Moreover, a Stx2 neutralizing antibody has been reported to protect mice against weight loss and death by reducing EHEC colonization [85]. These studies suggest that this toxin is the single substance involved in promoting bacterial intestinal infections and exacerbating disease. In addition, Stx1 was found to modulate the expression of galetin-3, which is associated with sodium absorption in the intestine and may contribute to diarrhea [86]. The Stx-induced inflammatory responses of IECs may enhance colonization by EHECs [55,87]. Compared with homologous mutations without the Stx gene in rabbit colon epithelium, wild-type EHEC modulates more diverse transcriptome responses and regulates cytokine gene expression [88].

Stxs stimulate the release of pro-inflammatory cytokines by various host cell types, including those in the endothelium [89]. Serum concentrations of IL-8, MCP-1, and G-CSF have been reported to be higher in pediatric patients with HUS than in controls, with these concentrations associated with disease severity [90,91,92]. *E.* *coli* O157:H7 enteritis was associated with the production of GRO-α, MIP-1β, and MCP-1 in blood, regardless of the occurrence of HC or HUS [93]. STEC infection-associated alterations in the expression of cytokines and chemokines at specific cellular levels is important for understanding the disease. A study assessing the effects of infection of IECs with STECs (97-3250: STEC O26: H11, 4865/96: STEC O145: H28) and HS (commensal *E. coli* O9:H4) on the expression of cytokine mRNA and protein found that 97-3250 promoted greater polymorphonuclear leukocytes (PMN) penetration than 4865/96 or HS by upregulating the expression of various chemokines, including CXCL8/IL-8, and by enhancing PMN chemotaxis. Moreover, of the strains tested, 97-3250 had the greatest effect on gene expression [60]. The presence of polymorphonucleocytes (PMNs) in the stool is considered a risk indicator for the development of HUS. Several invasive pathogens, including STEC, cause fever and inflammatory diarrhea, which is characterized by a high level of PMNs in stools [94]. Increases in the numbers of macrophages and leukocytes, as well as neutrophils, are associated with disease development [90,95].

## 4. Effects of Probiotics on STEC and Stxs in the Gut

**Figure 3:**
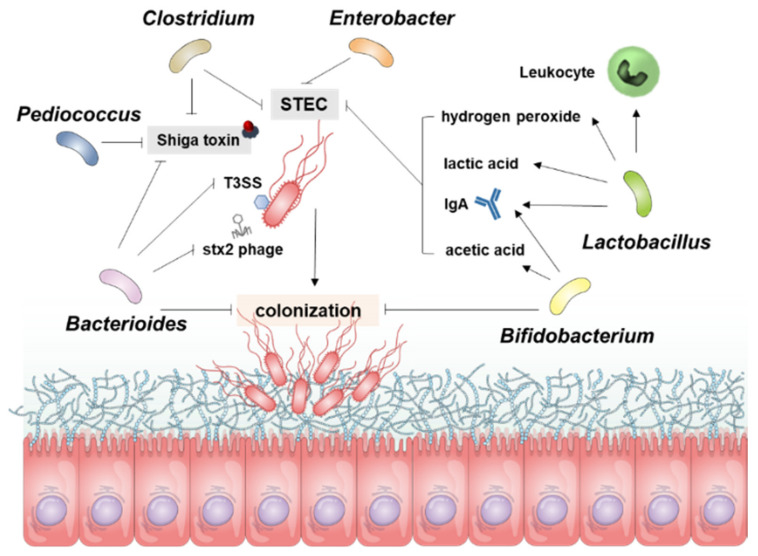
Overview for the Resistance Mechanism of gut microbiota to STEC Infection. The resistance of microbial guns to control STEC works in several ways, and the diagram shows the role of each bacterium in STEC. *Bacterioides* inhibit Stx production, control direct bacteria and inhibit the colonization of STEC. *Bifidobacterium* also inhibits STEC colonization and controls STEC proliferation through acetic acid and IgA. *Lactobacillus* is involved in inhibiting STEC proliferation through the production of hydrogen peroxide, lactic acid, IgA, and leukocyte activity. *Pediococcus*, *Clostridium*, and *Enterobacter* are involved in inhibiting STEC proliferation or controlling Stx production.

*E. coli* is mainly found in the intestinal cecum and colon of mammals and resides in the mucosal layer, from which it moves into the intestinal lumen and is excreted into the feces. Pathogenic *E. coli*-mediated diseases, such as food poisoning, intestinal tissue damage, and particularly bloody diarrhea in young children, are major concerns in public health and medical expense-related economic problems worldwide. More importantly, certain strains of STEC in the gut may cause severe extraintestinal or extrarenal illnesses in humans. Certain Gb_3_-expressing cell types in the gut, such as Paneth cells, may serve as portals for ingress of Stxs. Several epidemiological studies have shown that infection with STEC isolates expressing Stx2a is more pathogenic than infection with strains producing Stx1a or Stx1a+Stx2a [4,33,36]. Infection with strains producing Stx2a may lead to extraintestinal complications, although the cause of the latter is likely to be multi-factorial and can include the constitutive regulation of *stx* gene expression; antibiotic usage during the prodromal diarrheal phase, inducing the phage-mediated lytic cycle; the presence or absence of additional *E. coli* virulence factors; and variations in host responses to toxins, which all contribute to the outcome of infection. Moreover, *stx* genes are carried in the genomes of temperate phages [96,97], located in the late gene region downstream of the late promoters and upstream of the lysis cassette, highly expressed upon activation of phage-mediated lytic cycle and the toxin subunits assembled in the periplasm are secreted by the lytic cycle [98]. Therefore, antibiotics are not recommended for patients with HUS because they activate the phage-mediated lytic cycle in STEC [99] that lyses bacterial host cells to release Stxs and free phage particles that can infect other bacteria and transduce *stx* genes [100,101,102,103,104,105]. Given that antibiotic therapy is not indicated for these reasons, besides numerous therapeutic approaches utilizing Stxs-specific neutralizing antibodies, toxin receptor analogs, and vaccinations [106], studies have attempted to increase the population of intestinal microbiota that can inhibit STEC colonization and/or Stx expression (Table 1, Figure 3), thereby limiting HUS development.

### 4.1. Inhibitory Effects of Microbiota on STEC

#### 4.1.1. Inhibition of *E. coli* O157:H7 Growth

*Bacteroides* strains, which are abundant in human gut microbiota, are the most studied commensal bacteria against *E. coli* O157:H7. For instance, intestinal *E. coli* O157:H7 colonization was significantly lower in gnotobiotic mice pre-colonized with *Bacteroides fragilis* (*B. fragilis*) [107]. Due to the reduced colonization of *E. coli* O157:H7 in the intestines of mice pre-colonized with *B. fragilis*, the translocation of *E. coli* O157:H7 to other organs such as the kidneys, heart, liver, and spleen was significantly lower than in mice with non-commensal bacteria, leading to increased survival outcomes against *E. coli* O157:H7 [107]. Similar to *Bacteroides* strains, Lactobacilli such as *Lactobacillus reuteri* (*L. reuteri*) also have inhibitory effects against *E. coli* O157:H7 colonization independent of the antimicrobial compound reuterin, suggesting that *L. reuteri* has a direct role in protecting against EHEC [108]. The colonization of *E. coli* O157:H7 was decreased, and subsequently, the necrosis of the kidneys and weight loss were significantly ameliorated in *L. reuteri*-fed mice [108]. Cattle that were fed *Lactobacillus acidophilus* (*L. acidophilus*) also showed the inhibition of *E. coli* O157:H7 colonization in the feces [109].

In addition to the direct role of microbiota against the colonization of *E. coli* O157:H7, indirect roles such as the secretion of molecules, modulation of pH, and competition for nutrients have been reported to suppress the growth of EHEC. For instance, intestinal N-acetylglucosamine (NAG), which is derived from the degradation of commensals such as *Bacteroides thetaiotaomicron* (*B. thetaiotaomicron*) by mucin, inhibits the colonization of BALB/c mouse intestines by *E. coli* O157:H7 and represses the expression of T3SS-encoding genes [110]. Reuterin, which was converted from glycerol by *L. reuteri*, completely suppressed the growth of *E. coli* O157:H7 incubated in bovine rumen fluid [111]. *L. acidophilus* strain La-5 was also found to control the transcription of *E. coli* O157:H7 genes associated with colonization by secreting molecules that act as QS signal inhibitors or interact directly with regulators of bacterial gene transcription [112]. Hydrogen peroxide produced by *Lactobacillus lactis* [113] and lactic acid produced by *Lactobacillus* strains [136] were found to significantly reduce the colonization of *E. coli* O157:H7, the latter by altering pH [114]. Likewise, yoghurt containing *Bifidobacterium bifidum* (*B. bifidum*) was found to inhibit colonization by *E. coli* O157:H7, followed by a reduction in pH [115]. Mice administrated *E. coli* O157:H7 and the acetic acid-producing *Bifidobacterium breve* (*B. breve*) show significantly reduced amounts of *E. coli* O157:H7 in the feces, leading to higher survival rates and body weight than mice administrated *E. coli* O157:H7 alone, suggesting that the acetate secreted by *B. breve* lowered the pH in the intestines and thus inhibited disease pathogenesis [116]. Butyrate-producing *Clostridium butyricum* (*C. butyricum*) inhibits the growth of *E. coli* O157:H7 and reduces its lethality in gnotobiotic mice [78]. Another novel butyrate-producing bacteria, *Anaerostipes butyraticus*, was found in low-*E. coli* O157:H7-shedding calves and cattle, suggesting that butyrate-producing bacteria in the GI tract can be used to treat *E. coli* O157:H7 infection [137]. Similarly, other butyrate-producing bacteria, such as strains of *Porphyromonadaceae* [138], *Lachnospiraceae* [139], *Ruminococcaceae* [140], and *Clostridium sartagoforme* [141], increased with age in cattle, suggesting a relationship between the attachment/shedding of *E. coli* O157:H7 and butyrate-producing bacteria [137]. Competition for nutrients by probiotics was reported to inhibit the colonization of *E. coli* O157:H7. *Enterobacter asburiae* reduced *E. coli* O157:H7 survival 20- to 30-fold on lettuce by competition for carbon and nitrogen substrates [117].

#### 4.1.2. Regulation of the Host Immune System

Commensal microbiota not only inhibit *E. coli* O157:H7 growth but also regulate host immune responses to reduce the pathogenesis of HUS. For example, mice infected with *E. coli* O157:H7 and fed *Lactobacillus rhamnosus* (*L. rhamnosus*) HN001 show increased intestinal anti-*E. coli* IgA responses [120]. In addition, blood leukocyte activity was higher in *L. rhamnosus* HN001-fed mice than in controls, leading to the decreased translocation of *E. coli* O157:H7 and associated lethality [120]. Similarly, infant rabbits administered *Lactobacillus casei* (*L. casei*) and infected with *E. coli* O157:H7 show increased levels of IgAs against Stx1, Stx2, and *E. coli* O157:H7 in the intestines [118]. *L. casei*-fed infant rabbits show decreased diarrhea, damage of the intestinal mucus, and colonization of *E. coli* O157:H7 independent of pH and fermented products such as lactic acid, suggesting that *L. casei* enhances local immune responses to *E. coli* O157:H7 [118]. Similar to Lactobacilli, Bifidobacteria also enhance the host immune response to *E. coli* O157:H7. The proportions of phagocytically active cells in the blood and peritoneum were significantly higher in *Bifidobacterium lactis* (*B. lactis*)-fed mice infected with *E. coli* O157:H7 than in controls [121]. In addition, the level of intestinal IgA against *E. coli* O157:H7 was higher in *B. lactis*-fed mice than in controls, which may help to inhibit the translocation of *E. coli* O157:H7 [121]. The levels of serum IgG and IgM, intestinal IgA antibodies against *E. coli* O157:H7, were also higher and the levels of fecal *E. coli* O157:H7 and intestinal injuries were lower in *Bifidobacterium thermacidophilum*-fed mice than in control mice, leading to decreased lethality [119].

#### 4.1.3. Reduction of Stx Production, Gene Expression, and Stx Phage Particle Release

*B. thetaiotaomicron* produces a soluble factor with a molecular weight <3 kDa that inhibits the SOS response of *E. coli* O157:H7 mediated by RecA and consequently inhibits *Stx2* phage particle release and Stx2 synthesis independent of pH [122]. *B. thetaiotaomicron* also inhibited the production of Stx2 by *E.* *coli* O157:H7 via uptake of vitamin B_12_ [123]. Interestingly, mutated *B. thetaiotaomicron*, which does not express an outer membrane receptor for vitamin B_12_, did not significantly inhibit the production of Stx2 by *E. coli* O157:H7, suggesting that vitamin B_12_ is essential for activating the LEE operon of the latter [123]. *B. thetaiotaomicron* also inhibits *Stx* phage production in *E. coli* O153:H25, which may reduce the production of Stxs [124]. Moreover, the reduction of the pH due to the production of organic acids by probiotics such as *Pediococcus pentosaceus*, *L. rhamnosus* GG, and *Bifidobacterium thermophilum* reduces the gene expression of Stx2 in *E. coli* O157:H7 [125]. *B. fragilis*-fed gnotobiotic mice also have a decreased level of Stxs in their feces, leading to reduced lethality [107]. *B.* *breve*, which reduces lethality in mice infected with *E. coli* O157:H7, inhibits Stx production due to a reduced intestinal pH and a higher concentration of acetic acid [116]. *Bifidobacterium infantis* and *Bifidobacterium longum* also reduce production of Stxs in the intestines of gnotobiotic mice [75]. *C. butyricum*, which reduces lethality induced by *E. coli* O157:H7 in gnotobiotic mice, decreases the fecal level of Stxs [78]. *L. casei*-fed infant rabbits demonstrated decreased level of Stxs in the large intestines due to anti-Stx1 and -Stx2 IgA antibodies in the colon [118].

#### 4.1.4. Reduction of A/E Lesions

Interestingly, it is reported that *Lactobacillus* strains reduce the pathogenesis of *E. coli* O157:H7 by reducing A/E lesions. For instance, the cell-free spent medium (CFSM) of *L. acidophilus* decreased attachment of *E. coli* O157:H7 to epithelial cells in vitro such as HeLa and HEp-2 cells, suggesting that the CFSM may block QS mechanisms in EHEC [126]. The biologically active fraction of the CFSM of *L. acidophilus* also reduces attachment of *E. coli* O157:H7 to the intestinal epithelium of ICR mice and subsequently decreases body weight loss [126]. Similar to the CFSM of Lactobacilli, the surface-layer protein extracts of *Lactobacillus helveticus* (*L. helveticus*) decreased the A/E lesions of *E. coli* O157:H7 and preserved the barrier function of the monolayers of HEp-2 and T84 cells in vitro [127]. In addition, *L. rhamnosus* inhibits the adhesion of *E. coli* O157:H7 to Hep-2 and T84 cells by adhering to these cells, thereby reducing the A/E lesions of *E. coli* O157:H7 [128].

### 4.2. Enhancing Effects of Microbiota on STEC

#### 4.2.1. Enhancement of Stxs Production

It is reported that the incubation of nonpathogenic phage-susceptible *E.* *coli* with toxin-encoding phages resulted in up to a 40-fold greater production of toxin when compared with lysogens alone, suggesting that nonpathogenic phage-susceptible commensal *E. coli* may play a role in the pathogenesis of HUS [129]. Similarly, intestinal Stx production was upregulated in CD-1 mice colonized with nonpathogenic phage-susceptible *E. coli* after infection with *E. coli* O157:H7 [130]. Moreover, the DNase colicins (E8/9) produced by some strains of nonpathogenic *E. coli* were found to increase Stx2 production from 8- to 64-fold compared with controls via the activation of an SOS response causing damage to *E. coli* O157:H7 DNA [131].

#### 4.2.2. Increased Expression of *E. coli* O157:H7 Virulence Genes

In addition to commensal strains of *E. coli*, strains of other probiotic species have been found to enhance the pathogenesis of *E. coli* O157:H7. *Bacteroides*, the abundant type of bacteria in human intestines [142], was found to enhance the virulence of *E. coli* O157:H7 as well as disease progression, although the inhibitory effects of *Bacteroides* against *E. coli* O157:H7 are well-known (Section 4.1). For instance, *B. thetaiotaomicron* increased the expression of *E. coli* O157:H7 virulence genes such as *ler*, the master LEE regulator, by regulating the transcription factor Cra, which is responsive to fluctuations in sugar concentrations [132]. Moreover, the in vitro addition of succinate, a major by-product of *Bacteroides* [143], increased the expression of the LEE-encoded protein EspA in *E. coli* O157:H7 but not in the *cra* mutant, suggesting an interplay between succinate and Cra [132]. Due to the increased expression of *E. coli* O157:H7 virulence genes, treatment of *B. thetaiotaomicron*-reconstituted C3H/HeJ mice with antibiotics resulted in significant weight loss following infection of *Citrobacter rodentium*, a natural mouse pathogen homologous to *E. coli* O157:H7 [132].

#### 4.2.3. Increased Colonization of *E. coli* O157:H7

Along with the secreted molecules such as succinate, the proteases secreted by *Bacteroides* were found to enhance the processing of the T3SS of *E. coli* O157:H7, increasing effector translocations and A/E lesion formation on host cells, which enhances the colonization of *E. coli* O157:H7 [133]. Furthermore, the fucose produced by *B. thetaiotaomicron* via multiple fucosidases that cleave fucose from host glycans in the intestines also enhances the colonization of *E. coli* O157:H7 [134]. A two-component signal transduction system named FusKR in *E. coli* O157:H7, in which FusK is the histidine sensor kinase and FusR is the response regulator, senses fucose and controls the expression of *E. coli* O157:H7 virulence genes, leading to robust colonization of *E. coli* O157:H7 [134].

#### 4.2.4. Increased Motility of *E. coli* O157:H7

Interestingly, a multi-omics study using an organ-on-a-chip microfluidic culture found that four human microbiome metabolites, 4-methyl benzoic acid, 3,4-dimethylbenzoic acid, hexanoic acid, and heptanoic acid, induced the expression of *E. coli* O157:H7 flagellin, which contributes to the pathogenesis of HUS [135]. These metabolites, however, did not alter colonization by *E. coli* O157:H7 or the concentration of Stx1, suggesting that metabolites derived from the human microbiome induce the pathogenesis of HUS, not by colonization and toxin production but by enhancing bacterial motility [135].

#### 4.2.5. Increased Expression of Toxin Receptors on Host Cells

Mice fed a HFD containing 10% guar gum showed elevated levels of butyrate, which is derived from fiber by their microbiota. In contrast to the ability of butyrate to inhibit the pathogenesis of *E. coli* O157:H7, the high concentrations of butyrate in HFD-fed mice were found to enhance *E. coli* O157:H7 colonization and lethality, as well as weight loss [45]. Elevated concentrations of butyrate produced by normal gut microbiota in HFD-mice also enhanced the expression of Gb_3_, the receptor of Stxs, suggesting that normal gut microbiota may indirectly enhance the pathogenesis of HUS [45].

## 5. Conclusions and Future Perspectives

Attempts to identify Stxs-induced risk factors in host cellular responses have revealed that these toxins have a wide range of novel properties that are associated with pathogenesis. Although studies have described Stx-induced signaling pathways that are associated with tissue damage, inflammation, and complement activation resulting from the immunopathological responses to these bacterial toxins, many details on the interfaces between STEC and commensal microbiota (Figure 3) remain to be determined. In particular, no coherent mechanism to date has defined the targets for intervention in HUS disease progression that might explain the dynamic immune modulation, or the mediators of inflammation associated with Stxs interacting with commensal gut microbiota. Additional studies are needed to better understand the intricate pathophysiology involving Stxs-associated bacterial communities in the gut. The precise mechanism by which changes of intestinal microbiota in response to EHEC Stxs may enhance host defense or exacerbate multi-organ damage warrants further investigation. These studies may help to identify potential targets for the disruption of innate immune responses or the protection of primary organs from damage induced by Stx1a and Stx2a.

## Figures and Tables

**Table 1:** Abilities of probiotics to regulate Stx production and STEC virulence.

Effect	Activity	Mediator	Model	Intestine In-/Out-Side	Genus	Species	Ref
Inhibition	Inhibit growth of *E. coli* O157:H7	Butyric, lactic acid	Gnotobiotic mice	Inside	*Clostridium*	*butyricum*	[78]
		N/D	Gnotobiotic mice	Inside	*Bacteroides*	*fragilis*	[107]
		N/D	Gnotobiotic mice	Inside	*Lactobacillus*	*reuteri*	[108]
		N/D	Cattle	Inside	*Lactobacillus*	*acidophilus*	[109]
		N-acetylglucosamine (NAG) and N-acetylneuraminic acid (NANA)	BALB/c mice Bovine rumen	Inside	*Bacteroides*	*thetaiotaomicron*	[110]
		Reuterin	fluid	Inside	*Lactobacillus*	*reuteri*	[111]
		Reduce autoinducer-2 (AI-2) production	*E. coli* O157:H7	N/D	*Lactobacillus*	*acidophilus*	[112]
		Hydrogen peroxide	Raw chicken meat	N/D	*Lactobacillus*	*lactis*	[113]
		Lactic acid	*E. coli* O157:H7	N/D	*Lactobacillus*	*casei*	[114]
		Decrease pH	*E. coli* O157:H7	N/D	*Bifidobacterium*	*bifidum*	[115]
		Acetic acid	BALB/c mice	Inside	*Bifidobacterium*	*breve*	[116]
		Nutrition competition (carbon, nitrogen)	Lettuce	N/D	*Enterobacter*	*asburiae*	[117]
		Production of anti-Stx1 and -Stx2 IgA in the colon	Infant rabbits	Inside	*Lactobacillus*	*casei*	[118]
		IgA	BALB/c mice	Inside	*Bifidobacterium*	*thermacidophilum*	[119]
	Regulate host immunity	Upregulate intestinal anti-*E. coli* IgA responses	BALB/c and C57BL/6 mice	Inside	*Lactobacillus*	*rhamnosus*	[120]
		Blood leukocyte activity					
		Inhibit translocation of *E. coli* O157:H7					
		Production of anti-Stx1 and -Stx2 IgA in the colon	Infant rabbits	Inside	*Lactobacillus*	*casei*	[118]
		Increase phagocytic activity	BALB/c and C57BL/6 mice	Outside	*Bifidobacterium*	*lactis*	[121]
		Increase production of IgA against *E. coli* O157:H7	BALB/c and C57BL/6 mice	Inside	*Bifidobacterium*	*lactis*	[121]
		Increase production of IgG and IgM against *E. coli* O157:H7	BALB/c mice	Outside	*Bifidobacterium*	*thermacidophilum*	[119]
		IgA	BALB/c mice	Inside	*Bifidobacterium*	*thermacidophilum*	[119]
	Reduce Stx production	N/D	Gnotobiotic mice	Inside	*Bifidobacterium*	*infantis*	[75]
		N/D	Gnotobiotic mice	Inside	*Bifidobacterium*	*longum*	[75]
		Butyric, lactic acid	Gnotobiotic mice	Inside	*Clostridium*	*butyricum*	[78]
		N/D	Gnotobiotic mice	Inside	*Bacteroides*	*fragilis*	[107]
		Acetic acid	BALB/c mice	Inside	*Bifidobacterium*	*breve*	[116]
		Production of anti-Stx1 and -Stx2 IgA in the colon	Infant rabbits	Inside	*Lactobacillus*	*casei*	[118]
		N/D	*E. coli* O157:H7	N/D	*Bacteroides*	*thetaiotaomicron*	[122]
		Uptake vitamin B_12_	*E. coli* O157:H7	N/D	*Bacteroides*	*thetaiotaomicron*	[123]
	Suppress kidney necrosis induced by *E. coli* O157:H7	N/D	Gnotobiotic mice	Inside	*Lactobacillus*	*reuteri*	[108]
	Repress T3SS of *E. coli* O157:H7	NANA and NAG	*E. coli* O157:H7	N/D	*Bacteroides*	*thetaiotaomicron*	[110]
	Reduce intestinal injuries after *E. coli* O157:H7 infection	Production of anti-Stx1 and -Stx2 IgA in the colon	Infant rabbits	Inside	*Lactobacillus*	*casei*	[118]
		Increased production of IgG and IgM against *E. coli* O157:H7	BALB/c mice	Inside	*Bifidobacterium*	*thermacidophilum*	[119]
	Inhibit *stx2* phage particle release	N/D	*E. coli* O157:H7	N/D	*Bacteroides*	*thetaiotaomicron*	[122]
	Reduce Stx2 gene expression	Inhibition of phage production	*E. coli* O153:H25	N/D	*Bacteroides*	*thetaiotaomicron*	[124]
		Organic acid produced by probiotics	*E. coli* O157:H7	N/D	*Pediococcus*	*pentosaceus*	[125]
					*Lactobacillus*	*rhamnosus GG*	
					*Bifidobacterium*	*thermophilum*	
	Reduce attaching and effacing lesions of *E. coli* O157:H7	Spent medium	ICR mice	Inside	*Lactobacillus*	*acidophilus*	[126]
			HeLa cells	N/D			
			Hep-2 cells				
		S-layer protein	Hep-2 cells	N/D	*Lactobacillus*	*helveticus*	[127]
			T84 cells				
		N/D	Hep-2 cells	N/D	*Lactobacillus*	*rhamnosus*	[128]
			T84 cells				
Enhancement	Increase toxin receptor expression on host cells	Butyrate	*E. coli* O157:H7	N/D	N/D	N/D	[45]
	Enhance toxin production	Bacteriophage transfer	CD-1 mice	Inside	*Escherichia*	*coli*	[129]
							[130]
		Damage of *E. coli* O157:H7 DNA using DNase colicins	*E. coli* O157:H7	N/D	*Escherichia*	*coli*	[131]
	Increase the expression of the virulence genes of *E. coli* O157:H7	Regulate Cra, a transcription factor for virulence genes of *E. coli* O157:H7	C3H/HeJ mice	Inside	*Bacteroides*	*thetaiotaomicron*	[132]
	Exacerbate weight loss after infection		*E. coli* O157:H7	Inside	*Bacteroides*	*thetaiotaomicron*	[132]
	Enhance colonization	Butyrate	*E. coli* O157:H7	N/D	N/D	N/D	[45]
		Secrete proteases that cleave the translocon of the T3SS	*E. coli* O157:H7	N/D	*Bacteroides*	*thetaiotaomicron*	[133]
		Fucose	*E. coli* O157:H7	N/D	*Bacteroides*	*thetaiotaomicron*	[134]
	Enhance T3SS of *E. coli* O157:H7	Secrete proteases that cleave the translocon of the T3SS	*E. coli* O157:H7	N/D	*Bacteroides*	*thetaiotaomicron*	[133]
	Increase *E. coli* O157:H7 motility	4-Methyl benzoic acid	*E. coli* O157:H7	N/D	N/D	N/D	[135]
		3,4-Dimethylbenzoic acid					
		Hexanoic acid					
		Heptanoic acid					

## Data Availability

Not applicable.
